# Supplementary material for: Histone H3G34R mutation causes replication stress, homologous recombination defects and genomic instability in S. pombe
Source: eLife. 2017 Jul 18;6:e27406. doi: 10.7554/eLife.27406 (PMC5515577; doi:10.7554/eLife.27406)
Supplement: Supplementary file 1. — DOI: http://dx.doi.org/10.7554/eLife.27406.017 [file elife-27406-supp1.docx]

**Supplementary File 1:** Peptides used for Antibody characterization and for mass spectrometry calibration.

| PP 33 | WT K36me2 | PATGGVK(me2)KPHRY-biotin |
| --- | --- | --- |
| PP 34 | WT K36me3 | PATGGVK(me3)KPHRY-biotin |
| PP 35 | WT K36 | PATGGVKKPHRY-biotin |
| PP 38 | G34R K36me2 | PATGRVK(me2)KPHRY-biotin |
| PP 39 | G34R K36me3 | PATGRVK(me3)KPHRY-biotin |
| PP 40 | G34R K36 | PATGRVKKPHRY-biotin |
| PP 42 | WT H3 cleavage | K(Ac)AAPATGGVK(Ac)K(Ac)PHR |
| PP 44 | G34R cleavage | K(Ac)AAPATGR |
| PP 45 | G34R cleavage | VK(Ac)K(Ac)PHR |
| PP 46 | G34R cleavage | VK(prop)K(prop)PHR |
| PP 52 | WT H3 cleavage | K(prop)AAPATGGVK(prop)K(prop)PHR |
| PP 54 | G34R cleavage | K(prop)AAPATGR |
| PP 55 | H3 cleavage | K(prop)AAPATGGVK(Ac)K(Ac)PHR |
