# Supplementary material for: Histone H3G34R mutation causes replication stress, homologous recombination defects and genomic instability in S. pombe
Source: eLife. 2017 Jul 18;6:e27406. doi: 10.7554/eLife.27406 (PMC5515577; doi:10.7554/eLife.27406)
Supplement: Supplementary file 2. — DOI: http://dx.doi.org/10.7554/eLife.27406.018 [file elife-27406-supp2.docx]

**Supplementary File 2.** Detection parameters of unique tryptic peptides from *S.* *pombe* histone H3

|  | Precursor ion (m/z) | Product ions (m/z) | Collision energy (eV) | Retention time (min) |
| --- | --- | --- | --- | --- |
| KpQLASKpAAR | 542.827 | 772.468 | 19 | 4.35 |
|  |  | 659.384 | 19 |  |
| KpQLASKaAAR | 535.819 | 758.452 | 19 | 3.50 |
|  |  | 645.368 | 19 |  |
| KaQLASKpAAR | 535.821 | 772.468 | 19 | 3.50 |
|  |  | 659.384 | 19 |  |
| KaQLASKaAAR | 528.812 | 758.452 | 19 | 2.45 |
|  |  | 645.368 | 19 |  |
| KpAAPATGGVKpKpPHR | 529.311 | 1259.722 | 22 | 5.25 |
|  |  | 933.563 | 22 |  |
|  |  | 593.352 | 22 |  |
| KpAAPATGGVKpKaPHR | 524.638 | 1245.706 | 22 | 4.85 |
|  |  | 919.547 | 22 |  |
|  |  | 579.336 | 22 |  |
| KpAAPATGGVKaKpPHR | 524.640 | 1245.706 | 22 | 4.85 |
|  |  | 919.547 | 22 |  |
|  |  | 593.352 | 22 |  |
| KaAAPATGGVKpKpPHR | 524.642 | 1259.722 | 22 | 4.85 |
|  |  | 933.563 | 22 |  |
|  |  | 593.352 | 22 |  |
| KpAAPATGGVKaKaPHR | 519.967 | 1231.691 | 22 | 4.35 |
|  |  | 905.532 | 22 |  |
|  |  | 579.336 | 22 |  |
| KaAAPATGGVKpKaPHR | 519.969 | 1245.706 | 22 | 4.35 |
|  |  | 919.547 | 22 |  |
|  |  | 579.336 | 22 |  |
| KaAAPATGGVKaKpPHR | 519.971 | 1245.706 | 22 | 4.35 |
|  |  | 919.547 | 22 |  |
|  |  | 593.352 | 22 |  |
| KaAAPATGGVKaKaPHR | 515.295 | 1231.691 | 22 | 3.80 |
|  |  | 905.532 | 22 |  |
|  |  | 579.336 | 22 |  |
| VTIQPKpDMQLAR | 485.938 | 1014.540 | 21 | 8.85 |
|  |  | 917.487 | 21 |  |
|  |  | 733.366 | 21 |  |
|  |  | 618.339 | 21 |  |
| VTIQPKaDMQLAR | 481.266 | 1000.524 | 21 | 8.75 |
|  |  | 903.472 | 21 |  |
|  |  | 733.366 | 21 |  |
|  |  | 618.339 | 21 |  |
| H3 G34R mutant |  |  |  |  |
| KpAAPATGR | 414.240 | 643.352 | 15 | 0.60 |
|  |  | 572.315 | 15 |  |
|  |  | 501.278 | 15 |  |
| KaAAPATGR | 407.233 | 643.352 | 15 | 0.45 |
|  |  | 572.315 | 15 |  |
|  |  | 501.278 | 15 |  |
| VKpKpPHR | 438.774 | 777.473 | 16 | 0.95 |
|  |  | 593.352 | 16 |  |
|  |  | 284.197 | 16 |  |
| VKpKaPHR | 431.767 | 763.457 | 16 | 0.70 |
|  |  | 579.336 | 16 |  |
|  |  | 284.197 | 16 |  |
| VKaKpPHR | 431.769 | 763.457 | 16 | 0.70 |
|  |  | 593.352 | 16 |  |
|  |  | 270.181 | 16 |  |
| VKaKaPHR | 424.759 | 749.442 | 16 | 0.40 |
|  |  | 579.336 | 16 |  |
|  |  | 270.181 | 16 |  |
