# Supplementary material for: Histone H3G34R mutation causes replication stress, homologous recombination defects and genomic instability in S. pombe
Source: eLife. 2017 Jul 18;6:e27406. doi: 10.7554/eLife.27406 (PMC5515577; doi:10.7554/eLife.27406)
Supplement: Supplementary file 4. — DOI: http://dx.doi.org/10.7554/eLife.27406.020 [file elife-27406-supp4.docx]

**Supplementary File 4:** *S.pombe* strains

| **Fig.** | **Strain** | **Genotype** |  |
| --- | --- | --- | --- |
| 1c,1d,1e,1g,1-S1b, 1-S1d, 1-S1e, 1-S1g,1-S1h, 1-S2, 2, 2-S1, 3b,4a,4b,4c, 4d, 5b,5e,6b,6c,6f, 6g, 6-S1b, d, 7a, 7c | PY 7062(h-), 7059(h+),1747 | H3.1/H4.1::his3^+^ H3.3/H4.3::arg3^+^ ade6-210 ura4D his3D arg3D | 1 |
| 1c, 1f, 1-S1c, d, e, g, 1-S2, 2, 2-S1 3b,4a,4b,4c,4d, 5b,5e,6b,6c,6f, 6g, 6-S1d, 7a, 7c | PY 7108(h-), 7662(h+) , 7107 | H3.2 G34R H3.1/H4.1::his3 H3.3/H4.3::arg3 ade6-210 ura4D his3D arg3D |  |
| 1c, 1f, 1-S1e, S1g, 2, 2-S1,3b,4b,4c,4d, 5b,6f,6g, 6-S1b | PY 9023, 9024 | h- set2∆::KanR H3.1/H4.1::his3 H3.3/H4.3::arg3 ade6-210 ura4D his3D arg3D |  |
| 1d, 1e, 1-S1h | PY 9135,9136 | h- set2::set2-3xFLAG-KanR H3.1/H4.1::his3 H3.3/H4.3::arg3 ade6-210 ura4D his3D arg3D | 4 |
| 1d, 1e, 1-S1h | PY 9138,9139 | h? set2::set2-3xFLAG-KanR H3.2 G34R H3.1/H4.1::his3 H3.3/H4.3::arg3 ade6-210 ura4D his3D arg3D |  |
| 1f, 1-S1h,4e | PY 10616, 10617 | h- H3.1/H4.1::his3 H3.3/H4.3::arg3 ade6-210 ura4-D18 his3D1 arg3D1 leu1-32 +plasmid(JP-466; pREP41 LEU2+ (empty vector)) |  |
| 1f, 1-S1h,4e | PY 10618, 10619 | h- H3.1/H4.1::his3 H3.3/H4.3::arg3 ade6-210 ura4-D18 his3D1 arg3D1 leu1-32 +plasmid(JP-2595; pREP41-Set2-3XFlag LEU2+) |  |
| 1f,4e | PY 10620, 10621 | h- H3.2 G34R H3.1/H4.1::his3 H3.3/H4.3::arg3 ade6-210 ura4 D-18 his3D1 arg3D1 leu1-32 +plasmid(JP-466; pREP41 LEU2+ (empty vector)) |  |
| 1f,4e | PY 10622, 10623 | h- H3.2 G34R H3.1/H4.1::his3 H3.3/H4.3::arg3 ade6-210 ura4 D-18 his3D1 arg3D1 Leu1-32 +plasmid(JP-2595; pREP41-Set2-3XFlag LEU2+) |  |
| 1f,4e | PY 10624, 10625 | h- set2∆::KanR H3.1/H4.1::his3 H3.3/H4.3::arg3 ade6-210 ura4-D18 his3D1 arg3D1 leu1-32 +plasmid(JP-466; pREP41 LEU2+ (empty vector)) |  |
| 1f, 4e | PY 10626, 10627 | h- set2∆::KanR H3.1/H4.1::his3 H3.3/H4.3::arg3 ade6-210 ura4-D18 his3D1 arg3D1 leu1-32 +plasmid(JP-2595; pREP41-Set2-3XFlag LEU2+) |  |
| 1f,4e | PY 10628, 10629 | h- set2∆::KanR H3.2 G34R H3.1/H4.1::his3 H3.3/H4.3::arg3 ade6-210 ura4-D18 his3D1 arg3D1, leu1-32 +plasmid(JP-466; pREP41 LEU2+ (empty vector)) |  |
| 1f, 4e | PY 10630, 10631 | h- set2∆::KanR H3.2 G34R H3.1/H4.1::his3 H3.3/H4.3::arg3 ade6-210 ura4-D18 his3D1 arg3D1, leu1-32 +plasmid(JP-2595; pREP41-Set2-3XFlag LEU2+) |  |
| 1-S1b, d, 4a, 5c, 6-S1b | PY 42 (h-) PY41(h+) | ade6-210 arg3-D4 his3-D1 leu1-32 ura4-D18 |  |
| 1-S1d | PY 624 | mis6(ts) leu1-32 ura4DS/E ade6-210? arg3D? his3D? | **2** |
| 1-S1d | PY 730 | h- nda3-km311 ade6-210 his1-102 leu1-32 ura4-DS/E | **3** |
| 1-S1h | PY 10612 | h- set2::set2-3XFLAG-KanR cen1::ura4? ade6-210 leu1-32 ura4D his3D arg3D +plasmid(JP-466; pREP41 LEU2+ (empty vector)) |  |
| 1-S1h | PY 10614 | h- set2::set2-3XFLAG-KanR cen1::ura4? ade6-210 leu1-32 ura4D his3D arg3D +plasmid (JP-2595; pREP41-Set2-3XFlag LEU2+) |  |
| 2-S1, 3b | PY 6344, 6345 | h- H3.1/H4.1::his3 H3.3/H4.3::arg3 clr4Δ::KanR ade6-210 arg3D his3D leu1-32? ura4D |  |
| 1-S2, 6-S2 | PY 10398 | h- cdc10-M17 H3.1/H4.1::his3+ H3.3/H4.3::arg3+ his3D arg3D ade6-704 leu1-32 ura4-D18 |  |
| 1-S2, 6-S2 | PY 10401 | h- cdc10-M17 H3.2 G34R H3.1/H4.1::his3+ H3.3/H4.3::arg3+ his3D arg3D ade6-704 leu1-32 ura4D18 |  |
| 1-S2 | PY 10376 | h- cdc25-22 H3.1/H4.1::his3+ H3.3/H4.3::arg3+ otr1R (dg-glu BamHI-Spe1 fragment) Sph1::ura4 ade6-210 ura4-D18 or DS/E his3D1 arg3D1 |  |
| 1-S2 | PY 10393 | h- cdc25-22 H3.2 G34R H3.1/H4.1::his3+ H3.3/H4.3::arg3+ otr1R (dg-glu BamHI-Spe1 fragment) Sph1::ura4 ade6-210 ura4-D18/DS/E his3D1 arg3D1 |  |
| 3a | PY 9140, 9142 | h- H3.1/H4.1::his3 H3.3/H4.3::arg3 [Ch16 ade6-216 m23::LEU2+] ade6-210 arg3D his3D leu1-32 ura4D |  |
| 3a | PY 9189, 9190 | h- H3.2 G34R H3.1/H4.1::his3 H3.3/H4.3::arg3 [Ch16 ade6-216 m23::LEU2+] ade6-210 arg3D his3D leu1-32 ura4D |  |
| 3a | PY 9740,9741 | h- set2∆::KanR H3.1/H4.1::his3 H3.3/H4.3::arg3 [Ch16 ade6-216 m23::LEU2+] ade6-210 arg3D his3D leu1-32 ura4D |  |
|  | PY 9529, 9530 | h- H3.1/H4.1::his3 H3.3/H4.3::arg3 [Ch16 ade6-216 m23::LEU2+] swi6D::arg3 ade6-210 arg3D his3D leu1-32 ura4D | 5 |
| 1-S2, 3c, 6-S2 | PY 10379, 10380 | h- nda3-km311 H3.1/H4.1::his3+ H3.3/H4.3::arg3+ ade6-210? (ura4-D18?) his3D1 arg3D1 |  |
| 1-S2, 3c, 6-S2 | PY 10382 | h- nda3-km311 H3.2 G34R H3.1/H4.1::his3+ H3.3/H4.3::arg3+ ade6-210? (ura4-D18?) his3D1 arg3D1 |  |
| 4a | PY 4503 | h? rad22A ura4D leu1-32 ade6-M210 can1-1 |  |
| 4b, 4c,6e,6f, 6-S1d | PY 9325, 9326 | h+/90? rhp51::KanR H3.1/H4.1::his3 H3.3/H4.3::arg3 ura4-D18 his3D arg3D leu1-32 |  |
| 2e, 4d,6f | PY 10316, 10317 | h- set2∆::KanR H3.2 G34R H3.1/H4.1::his3 H3.3/H4.3::arg3 ade6-210 ura4D his3D arg3D |  |
|  | PY 1798 | h- clr4D::KanR ade6-210 leu1-32 ura4DS/E arg3D his3D |  |
| 5c | PY 8717 | h- Chk1-3xHA H3.1/H4.1::his3+ H3.3/H4.3::arg+ leu1-32 ura4D his3D arg3D |  |
| 5c | PY 8723 | h- Chk1-3xHA H3.2 G34R H3.1/H4.1::his3+ H3.3/H4.3::arg+ leu1-32 ura4D his3D arg3D |  |
| 5c | PY 8620 | h- Chk1-3xHA ade6-M216 leu1-32 ura4D |  |
| 5d, 6g, 6-S1c | PY 8779h+,8780h- | rad11-GFP-KanR H3.1/H4.1::his3+ H3.3/H4.3::arg+ his3D arg3D ade6-704? leu1-32 |  |
| 5d, 6g, 6-S1c | PY 8784,8785 | h- rad11-GFP-KanR H3.2 G34R H3.1/H4.1::his3+ H3.3/H4.3::arg+ his3D arg3D ade6-704? leu1-32 ura4D |  |
| 5d | PY 10178 | h- cds1::ura rad11-GFP-KanR H3.1/H4.1::his3+ H3.3/H4.3::arg+ his3D arg3D ade6-704? leu1-32 ura4D |  |
| 5e | PY 8749,8750 | h- cds1::ura H3.2 H3.1/H4.1::his3+ H3.3/H4.3::arg+ leu1-32 ura4D his3D arg3D ade6M |  |
| 5e | PY 8745 | h+/90? chk1::ura H3.1/H4.1::his3+ H3.3/H4.3::arg+ leu1-32 ura4D his3D arg3D ade6M |  |
| 5e | PY 8731,8732 | h+/90? chk1::ura cds1::ura H3.1/H4.1::his3+ H3.3/H4.3::arg+ leu1-32 ura4D his3D arg3D ade6M |  |
| 5e | PY 8737 | h+/90? cds1::ura H3.2 G34R H3.1/H4.1::his3+ H3.3/H4.3::arg+ leu1-32 ura4D his3D arg3D ade6M |  |
| 5e | PY 8728 | h- chk1::ura H3.2 G34R H3.1/H4.1::his3+ H3.3/H4.3::arg+ leu1-32 ura4D his3D arg3D ade6M |  |
| 5e | PY 8741,8742 | h+/90? chk1::ura cds1::ura H3.2 G34R H3.1/H4.1::his3+ H3.3/H4.3::arg+ leu1-32 ura4D his3D arg3D ade6M |  |
| 6b | PY 8852,8853 | h- brc1::ura4 H3.1/H4.1::his3+ H3.3/H4.3::arg+ leu1-32 ura4D his3D arg3D ade6M |  |
| 6b | PY 9552,9553 | h- H3.2 orp1-4 his3D arg3D H3.1/H4.1::his3 H3.3/H4.3::arg3 leu1-32 ade6M ura4D |  |
| 6b | PY 9550,9551 | h- H3.2 G34R orp1-4 his3D arg3D H3.1/H4.1::his3 H3.3/H4.3::arg3 leu1-32 ade6M ura4D |  |
| 6b | PY 10534, 10535 | h- brc1::ura4 orp1-4 H3.1/H4.1::his3+ H3.3/H4.3::arg+ leu1-32 ura4-D18 his3D arg3D ade6M |  |
| 6c | PY 9523, 9525 | h- rhp18::ura4 H3.1/H4.1::his3 H3.3/H4.3::arg3 ura4-D18 leu1-32 ade6-704 or 210 his3d arg3d |  |
| 6c | PY 9528, A22 | h- rhp18::ura4 H3.2 G34R H3.1/H4.1::his3 H3.3/H4.3::arg3 ura4-D18 leu1-32 ade6-704 his3d arg3d |  |
| 6c | PY 9544, 9545 | h- ubc13::ura4 H3.1/H4.1::his3 H3.3/H4.3::arg3 ura4-D18 leu1-32 ade6-704 his3d arg3d |  |
| 6c | PY 9546, 9547 | h- ubc13::ura4 H3.2 G34R H3.1/H4.1::his3 H3.3/H4.3::arg3 ura4-D18 leu1-32 ade6-704 his3d arg3d |  |
| 6d | A116, A117 | ku70::ura H3.1/H4.1::his3 H3.3/H4.3::arg3 ade6-210 ura4 D-18 his3D1 arg3D1 leu1-32 |  |
| 6d | A118, A119 | ku70::ura H3.2 G34R H3.1/H4.1::his3 H3.3/H4.3::arg3 ade6-210 ura4 D-18 his3D1 arg3D1 leu1-32 |  |
| 6e | PY 9327, 9350 | rhp51::kanR H3.2 G34R H3.1/H4.1::his3 H3.3/H4.3::arg3 ade6-210 ura4-D18 his3D arg3D leu1-32 ade6-704? |  |
| 6g | PY 10597, 10598 | rad11-GFP-KanR set2∆::KanR H3.1/H4.1::his3+ H3.3/H4.3::arg+ his3D arg3D ade6-704? leu1-32? ura4-D18 ade6-210? |  |
| 6g, 6-S1c, d | PY 8773, 8774 | h- rad22-YFP-kanMx4 H3.1/H4.1::his3+ H3.3/H4.3::arg+ his3D arg3D ade6M ura4-D18 leu1-32 |  |
| 6g, 6-S1c, 7d | PY 8781, 8782, 8783 | rad22-YFP-kanR H3.2 G34R H3.1/H4.1::his3+ H3.3/H4.3::arg+ his3D arg3D ade6M ura4D leu1-32 |  |
| 6g | PY 10600, 10601 | h+/90? set2∆::KanR rad22-YFP-kanMx4 H3.1/H4.1::his3+ H3.3/H4.3::arg+ his3D arg3D ade6M ura4-D18 leu1-32 |  |
| 6-S1c | PY A179 | h- set2::Kan MX ade6-210 leu1-32 ura4-D18 | **7** |
| 6-S1c | PY A180 | h+ set2::Kan MX ade6-210 leu1-32 ura4-D18 | **7** |
| 6-S1c | PY A178 | h-set2::ura ade6-210 arg3-D4 his3-D1 leu1-32 ura4-D18 | **7** |
| 6-S1c | PY 10571 | h? Ku70::ura ade6-210 otr1R (sph1):ade6+ leu1-32 | **8** |
| 6-S1c | PY 10572 | h? Ku70::ura ade6-210 otr1R (sph1):ade6+ leu1-32 | **8** |
| 6-S1b | PY 4392 | h+/90 set2∆::KanR ade6-210 arg3-D4 his3-D1 leu1-32 ura4-D18 |  |
| 6-S1e | PY 9027, 9028 | rad11-GFP-KanR rad22-RFP-kanR H3.1/H4.1::his3+ H3.3/H4.3::arg+ his3D arg3D ade6-704? leu1-32 ura4D |  |
| 6-S1e | PY 9044,9045 | rad11-GFP-KanR rad22-RFP-kanR H3.2 G34R H3.1/H4.1::his3+ H3.3/H4.3::arg+ his3D arg3D ade6-704? leu1-32 ura4D |  |
| 6-S3d | PY A189 | h- H3.2 G34R rad11-GFP-KanR cdc10-M17 H3.1/H4.1::his3+ H3.3/H4.3::arg3+ his3D arg3D ade6-704 leu1-32 ura4-d18 |  |
| 7a | PY 8618 | h- cds1::ura4 ura4D leu1-32 |  |
| 7b | PY 10360,10361 | h+/90 leu1-32 :: hENT1-leu1 + (pJAH29) his7-366:: hsv-tk-his7+ (pJAH31)  H3.1/H4.1::his3 H3.3/H4.3::arg3 ade6-210 ura4D his3D arg3D | **6** |
| 7b | PY 10363,10364 | h+ leu1-32 :: hENT1-leu1+ (pJAH29) his7-366:: hsv-tk-his7+ (pJAH31)  H3.2 G34R H3.1/H4.1::his3 H3.3/H4.3::arg3 ade6-210 ura4D his3D arg3D |  |
| 7d | PY 8773,8774 | h- rad22-YFP-kanR H3.1/H4.1::his3+ H3.3/H4.3::arg+ his3D arg3D ade6M ura4D leu1-32 |  |

Strains derived from: 1(Mellone et al., 2003), 2(Saitoh et al., 1997), 3(Hiraoka et al., 1984), 4(Morris et al., 2005), 5 (Creamer et al., 2014), 6 (Hodson et al., 2003), 7 (Pai et al., 2014), 8 (Manolis et al., 2001).
