# Supplementary material for: Histone H3G34R mutation causes replication stress, homologous recombination defects and genomic instability in S. pombe
Source: eLife. 2017 Jul 18;6:e27406. doi: 10.7554/eLife.27406 (PMC5515577; doi:10.7554/eLife.27406)
Supplement: Supplementary file 5. — DOI: http://dx.doi.org/10.7554/eLife.27406.021 [file elife-27406-supp5.docx]

**Supplementary File 5:** Primers used for real time PCR analyses.

| JPO-816 | *tlh1* real-time primer | 5'-CGTTTTTGATACCGGCGC-3' |
| --- | --- | --- |
| JPO-819 | *tlh1* real-time primer | 5’-TTGCCGTAACGACATCATGG-3’ |
| JPO-793 | *adh1* real-time primer | 5'-AACGTCAAGTTCGAGGAAGTCC-3' |
| JPO-794 | *adh1* real-time primer | 5'-AGAGCGTGTAAATCGGTGTGG-3' |
| JPO-769 | *cen dh* real-time primer | 5'-CCAGACCATTACAAGCACTACATACG-3' |
| JPO-770 | *cen dh* real-time primer | 5'-GAATCTTCTCTTGAATAAAACCGCC-3' |
| JPO-986 | *cen dg* real-time primer | 5'-GATACTGATAATATTGAGATCCACAGCAC-3' |
| JPO-987 | *cen dg* real-time primer | 5'-GCGATGCCAAACAACAATATTG-3' |
| JPO-2241 | *cen imr* real time primer | 5’-TGAAGGCTGTTGATTTGTGG-3’ |
| JPO-2242 | *cen imr* real time primer | 5’-ACAAACTGTTTTTGTTCAACGATT-3’ |
| JPO-2000 | *act1^+^* real time primer | 5’-AACCCTCAGCTTTGGGTCTT-3’ |
| JPO-2001 | *act1^+^* real time primer | 5’-TTTGCATACGATCGGCAATA-3’ |
| JPO-1693 | *clr4*^+^ real time primer | 5’-CAAAGAAGCTGGGGAAGACGA-3’ |
| JPO-1694 | *clr4*^+^ real time primer | 5’-GAAGGGTTTCGCGGTTTTGT-3’ |
| WB11 | q-cnt2-fl | 5’-CATTAAACAAACAACGGCACAC-3’ |
| WB12 | q-cnt2-fl | 5’-TAAGCCAGCAAATTCCTTGAG-3’ |
| JPO-4362 | *fah1^+^* real time primer | 5’-ATTGCACCTATTTGGGCATC-3’ |
| JPO-4363 | *fah1^+^* real time primer | 5’- GCTGACATCGGACAAATCAA-3’ |
| JPO-4364 | *grt1^+^* real time primer | 5’- CACCGCCCTTTCGTACTTAT-3’ |
| JPO-4365 | *grt1^+^* real time primer | 5’- TCCAATAACCTTCCCACTGC-3’ |
